# Targeted Immunotherapies in Gastrointestinal Cancer: From Molecular Mechanisms to Implications

**DOI:** 10.3389/fimmu.2021.705999

**Published:** 2021-08-10

**Authors:** Ding-Kang Wang, Qian Zuo, Qing-Yu He, Bin Li

**Affiliations:** Ministry of Education (MOE), Key Laboratory of Tumor Molecular Biology and Key Laboratory of Functional Protein Research of Guangdong Higher Education Institutes, Institute of Life and Health Engineering, Jinan University, Guangzhou, China

**Keywords:** gastrointestinal cancer, colorectal cancer, gastric cancer, esophageal cancer, immune checkpoint inhibitor, immunotherapy

## Abstract

Gastrointestinal cancer is a leading cause of cancer-related mortality and remains a major challenge for cancer treatment. Despite the combined administration of modern surgical techniques and chemoradiotherapy (CRT), the overall 5-year survival rate of gastrointestinal cancer patients in advanced stage disease is less than 15%, due to rapid disease progression, metastasis, and CRT resistance. A better understanding of the mechanisms underlying cancer progression and optimized treatment strategies for gastrointestinal cancer are urgently needed. With increasing evidence highlighting the protective role of immune responses in cancer initiation and progression, immunotherapy has become a hot research topic in the integrative management of gastrointestinal cancer. Here, an overview of the molecular understanding of colorectal cancer, esophageal cancer and gastric cancer is provided. Subsequently, recently developed immunotherapy strategies, including immune checkpoint inhibitors, chimeric antigen receptor T cell therapies, tumor vaccines and therapies targeting other immune cells, have been described. Finally, the underlying mechanisms, fundamental research and clinical trials of each agent are discussed. Overall, this review summarizes recent advances and future directions for immunotherapy for patients with gastrointestinal malignancies.

## Introduction

Gastrointestinal (GI) cancers are among the top 10 most prevalent and deadliest tumors worldwide, accounting for 26% of global cancer incidence and 35% of all cancer-related deaths (1). To date, surgical resection remains the primary treatment option for patients with colorectal cancer (CRC), gastric cancer (GC) and esophageal cancer (EC). Despite advances in adjuvant and neoadjuvant chemoradiotherapy (CRT), a fair number of patients still develop distant metastases and therapy resistance (2). To improve the prognosis of GI cancers, new therapeutic strategies are urgently needed. Over the last few decades, immune-targeted therapy has emerged as a revolutionary option for cancer treatment (3); however, the regulatory role of the immune system underlying GI cancers remains to be clarified. Fortunately, with the development of immunotherapy, cancer immunotherapy mainly based on checkpoint inhibitors has shown great prospects in clinical research, which shows the importance of immunotherapy in cancer treatment.

In this review, we first provide an overview of GI in terms of the epidemiology, molecular pathogenesis and standard therapy regimens. In subsequent sections, we outline the functional and molecular basis of oncoimmunology, with an emphasis on novel immune checkpoint targets and examples of applications in both laboratory research and clinical trials. We hope that this review will bring new insight into cancer immunotherapy for oncologists and immunologists.

## Gastrointestinal Cancers: A General Overview

### Esophageal and Gastric Cancer

The esophagus and stomach are part of the upper GI tract, which is part of the digestive system. As two major types of EC, esophageal squamous cell carcinoma (ESCC) occurs more commonly in the upper or middle part of the esophagus, while esophageal adenocarcinoma (SCC) occurs in the lower part of the esophagus. GC can develop in any part of the stomach and can spread throughout the stomach and to other organs, such as the small intestines, lymph nodes, liver, pancreas and colon (4). EC and GC are listed as the seventh and fourth most prevalent cancers worldwide (5). Based on estimates from 2018, 36.4% of digestive cancers, including stomach, liver and esophageal cancers, in China have a very poor prognosis, and the 5-year overall survival (OS) rate is quite low (less than 35% from 2013 to 2015) (6).

In general, surgery plays a key role in the treatment of GC as well as EC at an early stage. Moreover, systemic therapy of advanced, metastatic esophageal and gastric cancer utilizes a combination of multiple cytotoxic chemotherapeutic agents. Combination chemotherapy with a platinum and fluoropyrimidine doublet, such as FOLFOX, CAPOX, cisplatin/5-fluorouracil (5-FU), or cisplatin/capecitabine, is a common regimen with the addition of trastuzumab for the treatment of HER2-positive disease (5, 7, 8). Other agents, such as irinotecan or taxanes, can be utilized with fluoropyrimidines, platinum and/or ramicurumab or applied as monotherapy for those unfit for combination regimens (9, 10).

### Colorectal Cancer

In recent decades, the incidence rate of CRC has shown an upward trend worldwide, especially in developing countries. In China, CRC has an incidence rate exceeding 14.2/100,000, a mortality rate exceeding 7.4/100,000 and a 5-year prevalence exceeding 52/100,000. In addition, the prevalence rate of CRC is obviously higher in senior citizens aged over 60 (11). Currently, traditional therapies for CRC include endoscopic and surgical local excision, downstaging preoperative radiotherapy and systemic therapy, extensive surgery for locoregional and metastatic disease, local ablative therapies for metastases, palliative chemotherapy, targeted therapy, and immunotherapy. Although these new treatment options have doubled the OS for advanced disease to 3 years, the best survival rates still occur in patients without metastasis.

## The Rationale for Immunotherapy in Gastrointestinal Cancer

**Figure 1:**
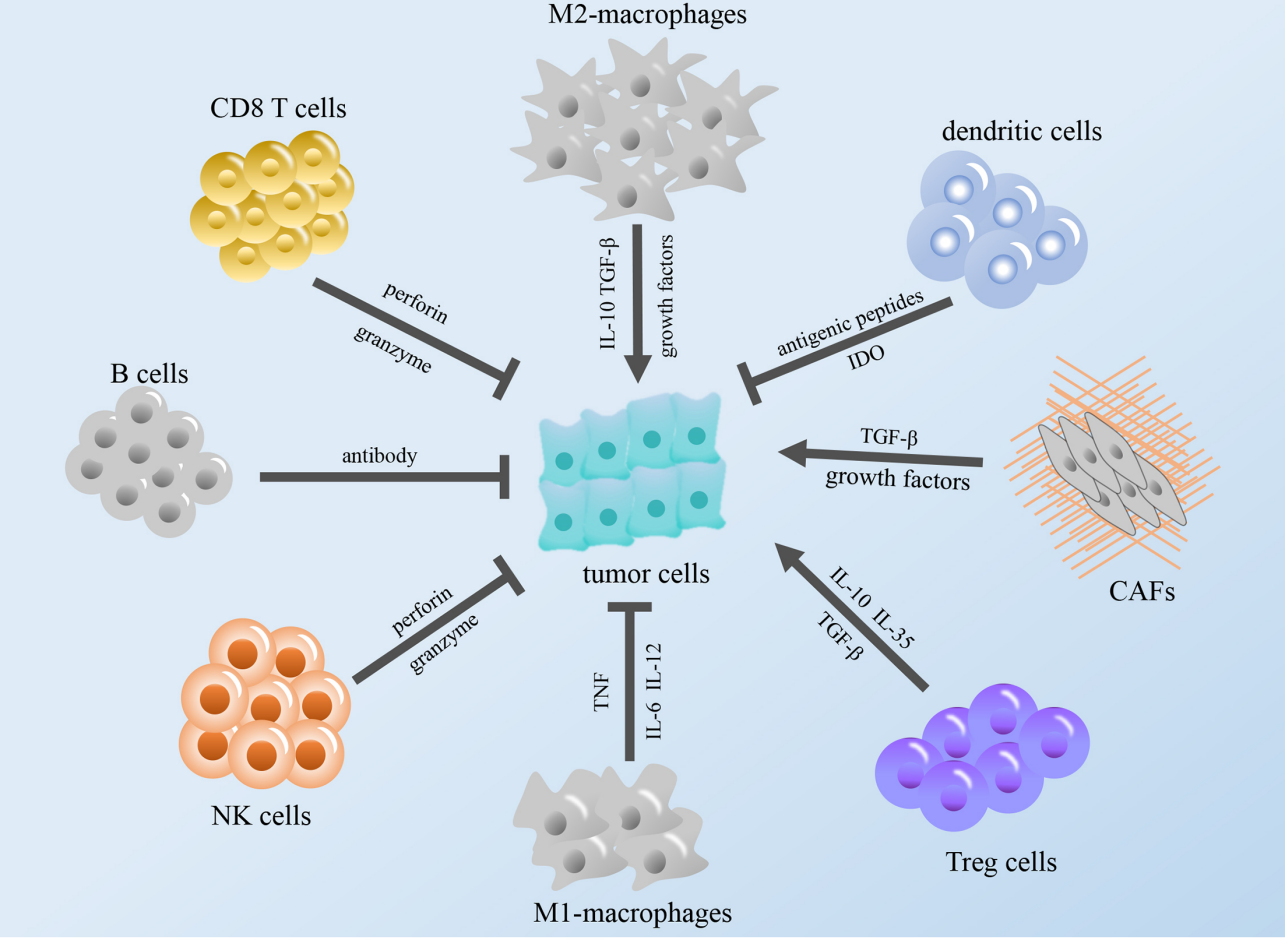
Modulation of the tumor immune microenvironment. The figure shows that immune cells in TME regulate tumor growth through cytokines and other regulatory factors. TGF-β, transforming growth factor β; IL, interleukin; NK cells, natural killer cells; TNF, tumor necrosis factor; IDO, indoleamine 2,3-dioxygenase; CAFs, cancer-associated fibroblasts; Treg cells, regulatory T cells.

The immune system exists within the body, and the execution of immune function is performed by the entire immune system. The immune system consists of immune organs, immune cells and immune molecules. These cell types surrounding cancer cells, including fibroblasts, endothelial cells, immune cells, and extracellular molecules, which include cytokines, hormones, cellular matrix, and growth factors, constitute the tumor microenvironment (TME) (12). According to relevant reports, some immune components in the TME can regulate the occurrence and development of tumors, and these components constitute the tumor immune microenvironment (TIME), which is expected to become a promising target for cancer immunotherapy (13–15) (**Figure 1**).

### Components of the TME

#### Tumor Cells

Although many improvements in molecularly directed therapies have been achieved, the prognosis of GI cancers remains poor (12). Breakthroughs in the immune checkpoint blockade offer potential therapeutic avenues, particularly with tools to overcome the mechanisms of immunosuppression in the TME. Currently, extensive publications highlight critical roles of the TIME in GI cancers, including CRC and GC (16, 17). As an important determinant of tumor progression and outcome in GI cancers, the TIME can shape cancer cell phenotypes and therapy responses through interplay with cancerous cells *via* chemokine and cytokine signaling or direct contact (18–20). Several reports have revealed that tumor progression and metastasis are subjected to not only genetic alterations within tumor cells but also the TIME elements. In brief, CD4+ T helper cells, CD8+ CTLs, NK cells, M1 macrophages, and DCs have been shown to be associated with a good prognosis (21). Conversely, CD4+ FOXP3+ Th2 cells, M2 macrophages, and myeloid-derived suppressor cells (MDSCs) have been attributed to a poor outcome (18).

#### Immune Cells

Tumor-infiltrating lymphocytes (TILs) are immune cells that have migrated to tumor tissue and the local microenvironment. This population is indicative of an immune response generated by the patient against the malignancy. TIL populations across GI tumors generally contain T lymphocytes, particularly CD8+ cytotoxic T lymphocytes (CTLs) (12). In EC cells, blocking the programmed cell death 1 (PD-1)/programmed cell death ligand 1 (PD-L1) and TGF-β signaling pathways can synergistically restore the function of antigen-specific CD8+ T cells and the capacity of antitumor T cells (22). In addition, functional MAGE-A3-specific CD8+ T cells have an independent prognostic effect on the survival of patients with ESCC (22). Recent studies have demonstrated that higher numbers of CD3+, CD8+, or CD45RO+ T cells in tumor tissue are significantly correlated with a superior disease outcome in patients with GC, and an imbalance in Th1 and Th2 cells can lead to an immunosuppressive state dominated by Th2-type cells (23). The Th1/Th2 cell ratio in peripheral blood in GC can be used to predict postoperative prognosis (24). Similarly, the type, density, and location of immune cells in CRC also have prognostic value that is superior to and independent of those of the tumor node metastasis (TNM) classification (25). In addition to T cells, there are many other immune cell types that infiltrate GI cancers.

Tregs, as a subtype of CD4+ T cells, can inhibit effector T cells *via* a series of chemokine signaling (26). FOXP3+ Tregs, a subtype of Tregs, their roles are ambiguous. Some studies have shown that a high density of FOXP3+ Tregs is beneficial to the prognosis of CRC after undergoing chemo or chemoimmunotherapy (27). On the other hand, it has been shown that Tregs in the esophageal mucosa and peripheral blood of patients with esophageal cancer increase significantly (28).

DCs, on the one hand, express MHC Class II and can present their antigenic peptides to CD4+ T cells. They activate effector T cells to attack tumors and play a crucial role in shaping the host response to cancerous cells. GC patients with good DC infiltration had lower lymph node metastases and lymphatic invasion and better 5-year survival rates (78%) than patients with less DC infiltration (29). On the other hand, activated DCs help in the expansion of Tregs, consequently leading to regulation of immune responses and thereby tumor immune escape (30). Meanwhile, DCs also stimulate the formation of M2 macrophages, thereby increasing the secretion of IL-10 and TGF-β (31), which reduces the expression of IL-12 expressed by DCs and inhibits the activation of adaptive responses (32).

Tissue-resident macrophages are present prior to the development of any malignancy (33, 34). Tumor-associated macrophages (TAMs) can differentiate into two distinct subtypes, M1 and M2. M1 macrophages secrete IL-6 and IL-12 to mitigate resistance during tumor development; they can also be activated by IFN-γ to secrete TNF to kill cancer cells, while M2 macrophages secrete growth factors that promote neoangiogenesis and tumor proliferation (35). In various types of cancers, increased numbers of TAMs are often related to a poor prognosis. However, the roles of TAMs in CRC remain controversial. According to some reports, on the one hand, a high density of TAMs predicts a better postoperative outcome (36), and on the other hand, TAMs also secrete cytokines that favor tumor development (37), which indicates that the impact of TAMs on CRC needs to be further explored. Additionally, in accordance with some studies, TGF-β and other growth factors secreted by cancer-associated fibroblasts (CAFs) promoted the proliferation of CRC cells through the Smad2/Smad4 pathway (38) and MAPK/PI3K/AKT pathway (39).

Neutrophils are similar to TAMs in classification. Neutrophils differentiate into N1 and N2 according to their polarization state. N1 has antitumor activity, which directly kill tumor cells by releasing reactive oxygen species (ROS) and reactive nitrogen species (RNS) (40, 41) and can also recruit M1 macrophages and promote T cell activation. In contrast, N2 has tumor-promoting activity, promoting angiogenesis and inhibiting the function of NK cells by releasing matrix metalloproteinase 9 (MMP9) (42). In addition, N2 recruits M2 macrophages and Treg cells and suppresses the function of CD8+ T cells, as well as other native neutrophils (43). Neutrophil extracellular traps (NETs) activate Toll-like receptor 9 on CRC cells through the MAPK pathway, which leads to the growth, migration and invasion of CRC cells (44).

B lymphocytes play an important role in tumor immunity and cancer biology. However, several studies have revealed that B lymphocytes can take part in carcinogenesis and tumor progression by producing antibodies to facilitate chronic inflammation in the TIME (45, 46). Moreover, regulatory B cells can also restrain antitumor responses mediated by T cells in cancer (47). Regulatory B cells (Breg cells), a novel subset of B lymphocytes, appear to facilitate tumor growth and progression *via* the production of IL-10 to suppress the activity of CD8+ T cells in squamous carcinoma (48). In CRC, the presence of B cells seems to be detrimental to prognostic outcome (49).

NK cells are unique in that they have both innate and adaptive immune properties. NK cells participate in the antitumor immune response through the production of proinflammatory cytokines, which recruit and induce the proliferation of other immune cells (12). Activated NK cells can directly kill some tumor cells and virus-infected cells. In EC, expanded NK cells had high cytotoxicity against ESCC cells, especially those with the epithelial-mesenchymal transition phenotype (50). In another investigation, a higher NK cell density was shown to be significantly related to a higher survival rate in GC, especially for advanced patients (51).

#### Cytokines/Chemokines

Cytokines are important players in the tumor microenvironment, and cytokines/chemokines are used to establish connections between various immune cells, such as IL-6 (52), IL-10 (53), IL-12 (54), IL-35 (55), epidermal growth factor (EGF), vascular endothelial growth factor (VEGF), tumor necrosis factor alpha (TNF-α) (56), interferon γ (IFN-γ) (57), indoleamine-2,3-dioxygenase (IDO) (58), and transforming growth factor beta (TGF-β) (59). For example, IL-10 and TGF-β switch macrophages from a M1-like (proinflammatory or classically activated) state to a M2-like (anti-inflammatory or alternatively activated) state. M1 macrophages induce antitumor immune signaling and correlate with tumor killing capacity. Conversely, M2 macrophages exhibit protumor effects and contribute to fibrosis and the production of matrix proteins (60) as well as angiogenesis, metastasis, and the suppression of adaptive immunity (61, 62). Human CRC cell lines cultured *in vitro* are able to polarize macrophages toward the M2 phenotype (63). In GC patient samples, M2 macrophages in the stroma may be correlated with the presence of a lesion (64). The CCL2-CCR2 axis in esophageal carcinogenesis contributes to immune evasion and tumor promotion through the PD-1 signaling pathway (65). At the same time, the activity of NK cells and effector T cells was suppressed due to the increased secretion of cytokines such as IL-10, TGF-β, EGF and VEGF (50).

#### Cells in the Extracellular Matrix (ECM)

Unlike strictly defined immune cells, fibroblasts are present within the stromal microenvironment and serve to produce extracellular matrix (ECM) proteins in particular collagen. They actively manufacture and respond to cytokines in cooperation with immune cells within the stromal microenvironment. Fibroblasts are also associated with epithelial cell polarity, proliferation, and, to some extent, tumorigenic potential. CAFs have been shown to drive increased tumor growth compared to normal fibroblasts (66). They contribute to cancer cell survival and progression *via* a series of nutrient-rich ECM proteins or ECM-degrading proteases, resulting in persistent chronic inflammation within the tumor microenvironment and enhanced epithelial mesenchymal transition (EMT) of tumor cells (67, 68).

Recent studies have demonstrated that CAFs have the capacity to produce proinflammatory cytokines, which disrupt the normal cytokine balance to stimulate tumor growth by initiating angiogenesis and inhibiting CTLs (69, 70). Additionally, CAFs have been shown to secrete high levels of the proinflammatory cytokines IL-1β, IL-8, IL-10, tumor necrosis factor-alpha (TNF-α), monocyte chemoattractant protein-1 (CCL2), stromal derived factor-1 (CXCL12) and interferon-beta (IFN-β) (71). The crucial role of CAFs in tumorigenesis has been addressed by genetic analyses showing that their gene expression profiles are very different from those of normal breast fibroblasts. Moreover, the expression profiles of CAFs obtained from tumors with poor versus good prognosis are also very different (72). Good-outcome fibroblasts were associated with immune modulators involved in the Th1 immune response. This includes the expression of T cell receptor complexes (CD8a, CD247, and CD3D), MHC class I protein binding and granzyme A/B activity. The poor-outcome stroma had increased levels of hypoxia and angiogenesis and decreased chemokines that stimulate NK migration and T cell survival (73).

### Mechanisms of Tumor Immune Escape

One role of the immune system in mediating tumorigenesis is called “cancer immune editing”, and it can be classified into 3 stages: elimination, stalemate, and escape. The elimination stage includes innate and adaptive immune responses to specific tumor-related antigens and is characterized by the effector functions of T cells, B cells and NK cells mediated by cytokines, including IFN-α, IFN-γ and IL-12. The stalemate stage of immune killing is mediated by the adaptive immune system and the persistence of a small number of malignant clones. The escape stage involves malignant clones gaining the ability to evade surveillance carried out by the adaptive immune system.

The understanding of the mechanisms of tumor immune escape changes with each passing day, and the established mechanisms are as follows. First, there can be a lack of a specific antigen or an alteration in antigen processing. In a study of SCC cell lines and tumor tissues, levels of MIR125a-5p and MIR148a-3p were found to be increased, reducing levels of ATP binding cassette subfamily B member 3 (TAP2) and MHC I, both of which are required for antigen presentation (74). Tumor cells lack the expression of major MHC I molecules or lose the intracellular processing mechanism, enabling the transfer of tumor antigens to the surface of tumor cells for T cells to recognize (75). The high expression of MHC I molecules in SCC is related to markers of the adaptive immune response and significantly decreased OS time in patients (74). Second, tumors can promote the formation of an immune-tolerant microenvironment by affecting the levels of cytokines, such as by increasing the secretion of IL-6, IL-10 and TGF-β or by consuming IL-2. The changes in these cytokines in EC cells promote the infiltration of Treg cells, MDSCs and other types of cells, thus suppressing the function of cytotoxic T cells (76). Third, tumors can upregulate the expression of PD-1 and PD-L1 to induce peripheral T cell depletion (77). Finally, many oncogenic cell signaling pathways were originally thought to be used only to accelerate cell division and growth, but they are now thought to be factors mediating immune escape. For example, constitutively activated KIT signaling in GI tumors can lead to overexpression of indoleamine-2,3-dioxygenase (IDO), thus increasing Treg cell infiltration and promoting tumor growth; furthermore, melanoma cells activated by β-catenin/Wnt signaling can inhibit DC-mediated antigen presentation and prevent CD8+ T cell infiltration (78).

## Immunotherapeutic Strategies in Gastrointestinal Cancer: The Current Scenario and Future Perspectives

Immunotherapy refers to the activation of the body’s immune system to fight against tumor cells. It manipulates the immune system to target cancer antigens or break the barriers of T cell infiltration. Immunotherapy methods mainly include cytokines, immune checkpoint inhibitors, CAR T cell treatments, tumor vaccines, and treatments involving other immune cells in the TME.

### Activating the Immune Response - Cytokines

Several cytokines impede cancer cell growth *via* direct antiproliferative or proapoptotic actions or by indirectly enhancing the cytotoxic effect of immune responses against cancer cells. For example, autocrine IL-10 activity on CD8+ T lymphocytes has been shown to be crucial for prolonging the effector activity of cytotoxic CD8+ T cells (79, 80). This concept has been evaluated in a phase I clinical trial in advanced treatment-refractory tumors (NCT02009449) using IL-10 conjugated with PEG to increase its half-life. The administration of PEGylated cytokines (termed pegilodecakin) resulted in partial responses in patients with uveal melanoma, renal cell carcinoma (RCC) and CRC (81). In addition, CCR5 is the receptor for both CCL3 and CCL5, while CCR2 binds to CCL2. In patients with metastatic CRC, CCR2/CCR5 inhibitors, coupled with either chemotherapy or nivolumab, suppressed myeloid cell recruitment by blocking the activity of these chemokines (82). Another paradigmatic case is IFN-α, first discovered in 1957, which induces antitumor efficacy by directly augmenting NK cell-mediated killing and acting on T and B lymphocytes to modulate their activity and/or survival.

### Immune Checkpoint Inhibitors

**Figure 2:**
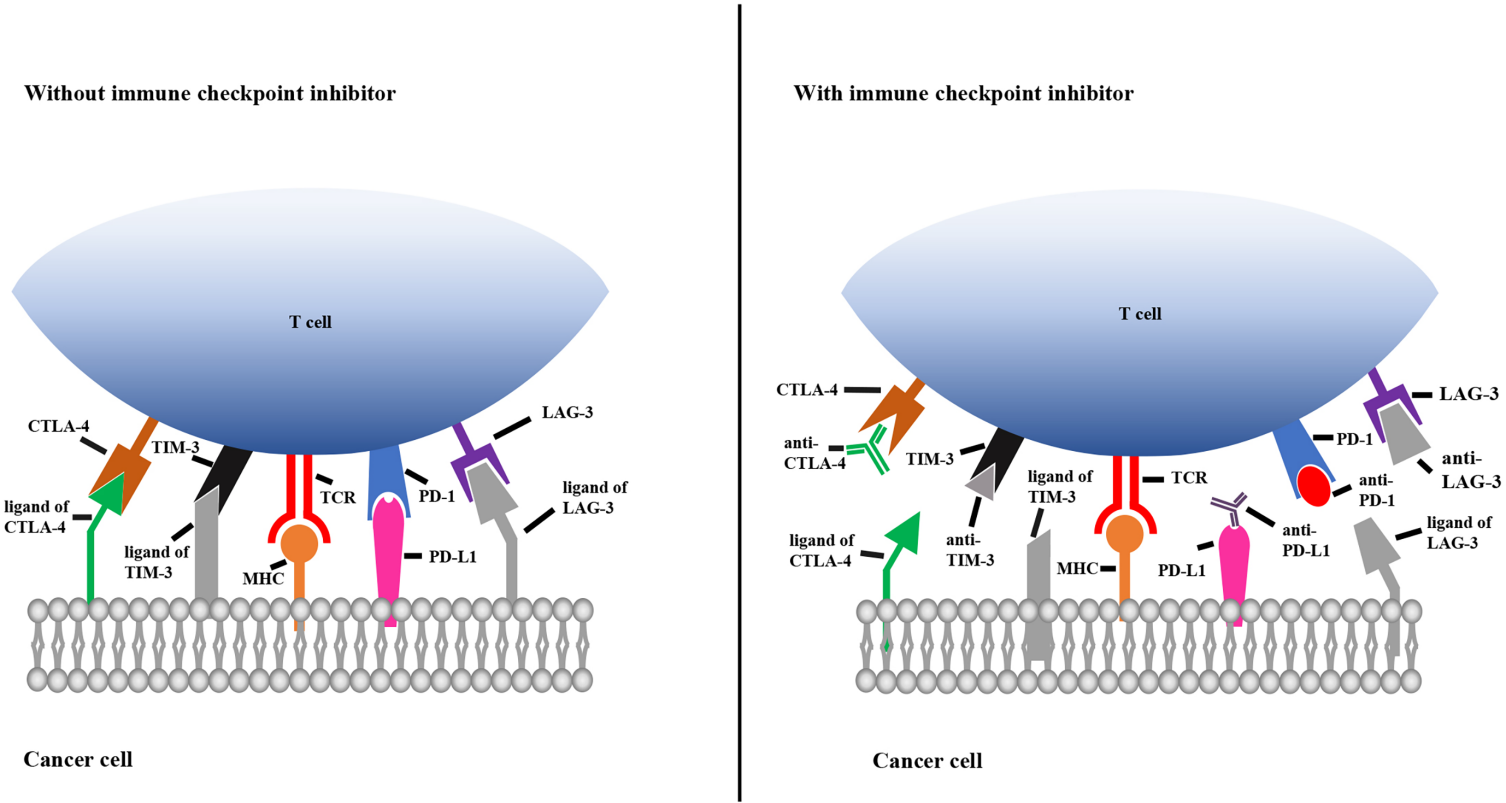
Mechanisms of immune checkpoint inhibitors. The left picture shows that the immune checkpoint molecules on T cells combine with the corresponding ligands on cancer cells, resulting in immunosuppression of T cell. The right picture shows that immune checkpoint molecules bind to the corresponding antibody to prevent T cell death. TCR, T cell receptor; MHC, major histocompatibility complex; TIM-3, T cell immunoglobulin 3; CTLA-4, cytotoxic T-lymphocyte-associated protein 4; PD-1, programmed cell death 1; PD-L1, programmed cell death ligand 1; LAG-3, lymphocyte activation gene 3.

Immune checkpoints play key roles in the innate immune system by ensuring that immune cells are capable of distinguishing self-antigens from exogenous antigens. In the TME, tumors often display self-antigens and escape immune surveillance. By blocking the interaction between immune cells and tumor cells expressing immune checkpoint molecules, checkpoint inhibitors allow the immune system to recognize tumor-associated antigens and consequently destroy malignant cells. As shown in **Figure 2**, when CTLA-4 and LAG-3 are bound by their corresponding monoclonal antibodies, T cells become activated and differentiate and proliferate; TIM-3 and PD-1 on T cells and PD-L1 on tumor cells are bound by their corresponding antibodies, which prevents T cell death, inhibits tumor cell evasion and promotes tumor cell apoptosis (**Figure 2**).

#### CTLA-4

As the first inhibitory receptor identified, CTLA-4 is mainly located on activated T lymphocytes and is also highly expressed in several types of cancer (83). There is a competitive interaction between CD28 and CTLA-4 in binding with CD80/CD86 molecules. CTLA-4 transmits an inhibitory signal to T cells, whereas CD28 transmits a stimulatory signal. A recent study revealed that the combined consideration of CTLA-4 expression and the platelet-lymphocyte ratio (PLR) has potential prognostic value for people suffering from ESCC (84). Therefore, it is not surprising that CTLA-4-targeted agents have shown great potential in the treatment of many cancers. In clinical trials, the tumor response rates of two anti-CTLA-4 agents, ipilimumab and tremelimumab, were approximately 10% (85). A large phase II clinical study explored the efficacy and safety of nivolumab ( ± ipilimumab) in the treatment of advanced CRC. The results revealed that most patients with microsatellite instability-high (MSI-H) metastatic CRC (mCRC) benefited from the nivolumab + ipilimumab regimen, and the regimen was well tolerated, but whether the combination therapy is superior to either drug as monotherapy therapy is still uncertain (86).

#### PD-1 and PD-L1/L2

PD-1 is one of the crucial immune checkpoint receptor proteins on T cells, B cells and NK cells and can bind to PD-L1 and/or PD-L2, which are located on the surface of multifarious tumor cells as well as hematopoietic cells. The combination of PD-1 and PD-L1/L2 expressed on tumor cells can dramatically inhibit the apoptosis of tumor cells, promoting the depletion of peripheral effector T cells and catalyzing the transformation of effector T cells into Treg cells (87). In 2017, the US Food and Drug Administration (FDA) approved pembrolizumab for the treatment of unresectable or metastatic MSI-H or mismatch repair deficient (dMMR) CRC that progressed following treatment with fluoropyrimidine, oxaliplatin, and irinotecan. This approval was based on results from the phase II CheckMate-142 trial (88), in which the objective response rate (ORR) was 28% in mCRC patients who received prior fluoropyrimidine, oxaliplatin, and irinotecan; in the trial, 1 patient had a complete response (CR), and 14 patients had partial responses (PRs). Furthermore, the expression of PD-L1 in cancer cells and the TME may contribute to the development of EBV-associated GC, and PD-L1 overexpression predicts large tumor size, lymph node metastasis, and poor prognosis in GC (89). A recent study presented at the American Society of Clinical Oncology (ASCO) meeting revealed that an anti-PD-1 monoclonal antibody could significantly prolong the OS of PD-L1-positive patients with advanced EC regardless of whether it was used alone or in combination with chemotherapy; furthermore, the antibody was highly safe (90, 91).

#### TIM-3

TIM-3 is a cell surface molecule expressed on DCs, monocytes, CD8 T cells and Th1 cells. The ligands of TIM-3 are frequently overexpressed in tumor cells. TIM-3 is a negative regulator of the antitumor immune response and has been proven to be associated with the occurrence and development of several malignant tumors. At both the mRNA and protein levels, the expression of TIM-3 in ESCC is remarkably higher than that in matched adjacent normal tissues; furthermore, researchers have found a close connection between the overexpression of TIM−3 and poor survival in patients with ESCC. Similarly, TIM-3 knockdown significantly inhibited the propagation, migration and invasion of ESCC cell lines. Further research explored whether the depletion of TIM-3 could restrain several signal transduction pathways, including the snail, p-GSK-3β and p-AKT pathways (92). In addition, some studies have suggested that the increased expression of TIM-3 on T cells may be partially responsible for the development of GC by inducing the secretion of IFN-γ and TNF-α (93). Another study has shown that reduced TIM-3 expression induced by genetic polymorphisms of TIM-3 may promote CRC invasion and metastasis (94). Taken together, these findings show that TIM-3 is a critical mediator in the progression of various cancers and may serve as a potential therapeutic target.

#### LAG-3

LAG-3 is another membrane protein expressed on B cells, some T cells, NK cells and TILs. LAG-3 can enhance the activation of Treg cells and prevent T cells from proliferating and differentiating into effector cells by binding with MHC II. In addition, some basic research has shown that dual blockade of LAG-3 and PD-1 can induce strong antitumor action (95). Furthermore, high expression of LAG-3 is associated with an improved survival rate in patients with ESCC or CRC and is being evaluated as a biomarker to predict response to antitumor treatment. These results indicate that LAG-3 can be used as a marker of the immune response for patients with ESCC or CRC (96, 97).

#### SIRPα

Signal regulatory protein-α (SIRPα) acts as an inhibitory receptor and is expressed on myeloid cells such as macrophages, DCs, mast cells and neutrophils. It interacts with the broadly expressed transmembrane protein CD47, also called the “don’t eat me” signal. In addition, the interaction between CD47 and SIRPα can help tumor cells avoid phagocytosis in the TME (98). The biological and preclinical relevance of the interaction of SIRPα and CD47 has been extensively investigated. A recent study showed that blocking SIRPα/CD47 signaling in ESCC cell lines could enhance the phagocytosis of ESCC cells in a dose-dependent manner (99). In addition, with the evolution of cancer immunotherapy, SIRPα/CD47 immunotherapy to stimulate the innate immune system as a cancer treatment has drawn increasing interest. Other reports have confirmed that the high expression of CD47 is an independent prognostic factor in GC, and the OS rate of patients with CD47-positive tumors was significantly lower than that of patients with CD47-negative tumors (100). Aberrant expression of CD47 has also been reported to strongly promote the proliferation of tumor cells (101).

### CAR T Cell Treatments

Chimeric antigen receptor (CAR) T cell therapy includes T cell modifications that enable activated T cells to recognize specific tumor cell surface antigens independent of MHC and eventually eliminate malignant cells (102). After many years of medical and laboratory research as well as specific clinical trials, in 2017, the US FDA approved the use of two CAR T cell therapies (103, 104). In recent years, some laboratories have attempted to exploit EphA2. CAR T cells recognizing EphA2 have demonstrated the capacity to identify and attack ESCC cells *in vitro*. These findings open a new avenue for future immunotherapies for ESCC (105). Coincidentally, CAR T cell therapy has been widely used in the treatment of other GI cancers and liver cancers. The HER2 gene plays an important role in the occurrence and development of GC, which highly expresses the protein p185; p185 shows negative expression in healthy individuals, and thus, it could be used as an ideal target for antitumor therapy with CAR T cells (106). Carcinoembryonic antigen (CEA), a sensitive tumor biomarker, was found to be a potent target for CAR T cell therapy for GI cancers (106). Therefore, a great deal of research has been carried out on the treatment of different cancers, such as liver cancer, cholangiocarcinoma and pancreatic cancer. Combined treatment integrating CAR T cell therapy with targeted therapies, such as therapies targeting CEA, HER2, GPC3, MUC1, CD133, EpCAM or EGFR, has exhibited great potential (107–110).

### Targeted Therapy Mediating Immune Cells

#### NK Cells

NK cells are innate lymphocytes and play a pivotal role in host immunity by killing virally infected and/or cancerous cells. Several clinical studies have suggested NK cell-based immunotherapy as a potential therapy for cancer. Inhibitory receptors, such as killer-cell immunoglobulin-like receptors (KIRs) and CD94/NK group 2 member A (NKG2A), recognize human leukocyte antigen (HLA) class I molecules on normal host cells. By lacking inhibitory receptor ligands, tumor cells that have downregulated surface MHC-I expression become susceptible to attack by NK cells. Current NK cell-based cancer immunotherapy aims to reverse tumor-induced NK cell dysfunction and sustain NK cell effector functions (111). To fight against tumors, NK cells can also be modulated to target the cell types responsible for maintaining the immunosuppressive TME, including M2-polarized macrophages, MDSCs, Treg cells, and fibroblasts (112). According to recent reports, adoptive cell therapy (ACT) has become a powerful tool to improve host antitumor activity (113), mainly including autologous NK cell transfer, allogeneic NK cell transfer and CAR-engineered NK cells (114). The three methods all artificially activate and expand NK cells *in vitro* and then transfer the modified NK cells to patients. Under the stimulation of some cytokines and costimulators, the antitumor ability of NK cells has been significantly improved (113, 115), and NK cells are expected to achieve staged success in the course of immunotherapy.

#### NKT Cells

Natural killer T cells (NKTs) are unique T lymphocytes that have the characteristics of conventional T cells and natural killer cells (116). They play an important role in connecting the natural immune system with the adaptive immune system and are also an important intermediary for mediating the immune response and tumor monitoring (117). It is usually composed of two parts, namely, the invariant TCRα chain and the semivariant TCRβ chain, recognizing the lipid antigen presented by the nonclassical MHC class I molecule CD1d (118). More importantly, the activation of CD1d-dependent invariant natural killer T cells (iNKT cells) can promote the release of cytokines such as IL-5, IL-6, IL-10, IL-17, IL-21, TNF-α, TGF-β and granulocyte-macrophage colony stimulating factor (GM-CSF) (116, 119, 120) and indirectly activate the antitumor mechanism. At present, several cancer immunotherapies based on iNKT cells have been developed, mainly including antigen-presenting cells (APCs) pulsed with α-GalCer, the transfer of *ex vivo*–expanded and/or activated iNKT cells, and iNKT cell–activating ligands (117). First, a clinical experimental study has not indicated any major toxicity or severe side effects in patients who have received repeated injection of the tumor immunity agonist α-GalCer (121), implying its clinical value in improving tumor immunity. In addition, patients with head and neck squamous cell carcinoma, who were treated with the combination of iNKT cells and α-GalCer-pulsed DCs, the antitumor immunity was greatly improved (122, 123)

#### Macrophages

Tumor-infiltrating immune cells play an ambiguous role in tumor development and progression. It has been extensively reported that TAMs promote or inhibit the expansion and dissemination of cancer cells depending on their functional states. Tumor-infiltrating macrophages are mainly recruited by CCL2 or CSF-1, and TAM depletion therapy (e.g., CCR2 or CSF1R antagonists) has already been tested in clinical trials for malignant solid tumors (124, 125). Some studies have also shown that CSF1R inhibitors represent a promising combination partner for T cell-enhancing immunotherapies (126, 127). Further investigation of the synergistic effects of these agents with immunotherapies will lead to the improvement of ongoing immunotherapeutic strategies. At the same time, some cancer immunotherapies targeting TAMs are on the rise, and most of them follow the principle of directly reducing the formation of TAMs or polarizing TAMs from the pro-tumor phenotype (M2-like), which helps tumor development to an antitumor phenotype (M1-like) (128). By preventing the recruitment of TAMs or blocking the CSF1/CSF1R axis, the survival rate of TAMs can be significantly reduced (129, 130), as mentioned above. In addition, some studies have shown that the polarization of TAMs to M1 macrophages can be promoted by targeting antibody-dependent cellular phagocytosis (ADCP) induced by the SIRPα/CD47 axis, thus enhancing the tumor immune response (131). Finally, a possible strategy for cancer treatment is the use of natural living macrophages as drug delivery vehicles (128).

#### γδT Cells

As a subtype of T lymphocytes, γδT cells have a wide range of antigen recognition abilities and can be used to recognize a series of antigens, including mitochondrial ATPase, human mutS homolog 2 and nonpeptide phosphoantigens (132–134), without using traditional APC to mediate antigen recognition (135, 136). The mechanism of antigen recognition depends on its peptide sequence. Since the sequence of complementarity determining region 3 (CDR3) of the delta chain is longer and more varied, it is more important in the process of antigen recognition (137). In addition to expressing NK cell receptors such as NGG2D, it may be a bridge between the innate immune system and adaptive immunity (138). According to related reports, under certain conditions, γδT lymphocytes may be activated in the late stage of the immune response (139, 140), which shows cytotoxicity by secreting perforin and granzyme and can also regulate the secretion of IFN-γ and TNF-α (141). Conversely, some cytokines may drive γδT lymphocytes to remodel tumor-immunosuppressive functions; thus, there is an urgent need to optimize γδT lymphocytes to obtain new antitumor targets (142). A recent study demonstrated for the first time that CAR-γδT cells obtained by modification using CAR technology are able to migrate to tumor cells and cross-express antigens (143), which opens a new avenue for cancer immunotherapy research.

#### IDO1

IDO1 is the rate-limiting enzyme that participates in tryptophan metabolism by converting the amino acid L-tryptophan (Trp) into L-kynurenine (Kyn). This enzyme assists tumor cells in maximally utilizing essential amino acids such as tryptophan in the TME to support tumor growth. However, as immune cells lose tryptophan, they lose their ability to fight cancer cells. Overexpression of IDO1 in tumor cells inhibits T cell function and impairs immune surveillance, leading to immune escape (144). In addition, it has been verified that the expression of IDO1 is associated with prognosis in EC and could be used as a prognostic biomarker (145). In a multivariate analysis, IDO1 expression was proven to be an independent prognostic factor for developing recurrence (146). Chen et al. reported that the enzyme activity of IDO1 directly affected the radiosensitivity of CRC, and IDO1 inhibition made CRC cells more sensitive to radiation-induced cell death and protected the normal small intestinal epithelium from radiation toxicity, indicating that IDO1 inhibition enhances the radiotherapy effect in CRC (147). According to relevant studies, IDO1 can also cooperate with COL12A1 to promote GC metastasis. This new discovery suggests that IDO1 and COL12A1 may be promising targets for anticancer therapy in GC (148).

### Tumor Vaccines

Tumor vaccines are a therapeutic method that can help educate the immune system to recognize cancer-related antigens and achieve antitumor effects. Tumor vaccines mainly include whole-cell vaccines, molecular vaccines and DC vaccines. DCs are one of the most effective APCs found in humans thus far. In addition, DC vaccines have achieved ideal results in clinical trials of malignant melanoma and prostate cancer (149). Due to the lack of MHC molecules and costimulatory molecules located on the surface of tumor cells, T lymphocyte immunity cannot be activated. Therefore, the immune response triggered by tumor vaccines mainly depends on the primary processing and further presentation of antigens by APCs, which is a key step in the development of effective adaptive immunity. As a new treatment strategy, immunotherapy based on tumor vaccines has received increasing attention in the treatment of advanced EC and other GI cancers (124, 150).

## Clinical Application of Immunotherapies in Gastrointestinal Cancers

### Colorectal Cancer

Immunotherapies have been studied extensively in CRC due to a better understanding of CRC subsets (particularly MSI/MMR status) and how they respond to immune modulation. Initial work on checkpoint inhibitors in CRC showed limited success. Neither the CTLA-4 inhibitor tremelimumab nor the PD-1 inhibitor nivolumab showed an objective response in CRC patients (151, 152). The Check-Mate-142 trial (phase II) evaluated single-agent nivolumab and nivolumab plus ipilimumab in patients with metastatic CRC with either MSI-H/dMMR or MSS/pMMR status. This study first investigated whether MSI-H tumors may be more responsive to immune checkpoint inhibitor therapy (88). Subsequent studies investigated targeting both CTLA-4 and PD-1 with ipilimumab/nivolumab in the pretreated population. The results were encouraging in that the ORR was 55%, the disease control rate for ≥ 12 weeks was 80%, and the progression-free survival (PFS) rates were 76 and 71% (9 and 12 months, respectively) (153). In the KEYNOTE-164 phase II trial, pembrolizumab monotherapy achieved an ORR of 28% and an excellent 1-year OS rate of 72% in heavily pretreated patients with MSI-H mCRC (154) responses to immune checkpoint inhibitors (155, 156).

Various regimens have been implemented to enhance immunogenicity in MSS/pMMR mCRC. The combination of atezolizumab and the MEK inhibitor conbimetinib showed limited effectiveness in CRC patients at advanced stages compared to regorafenib (157). A combination of FOLFOX and PD-1/PD-L1 inhibitors achieved an ORR of up to 50% and increased PFS (from 14 to 16 months) (158, 159). Preliminary data from the AVETUX trial demonstrated that a combination of FOLFOX, cetuximab, and the PD-L1 antibody avelumab showed a 75% ORR in patients with treatment-naïve microsatellite stable (MSS) mCRC (160). In contrast, the combination of atezolizumab (anti-PD-L1) with maintenance therapy with fluoropyrimidines and bevacizumab failed to improve the PFS of mCRC patients (161). In conclusion, all mCRCs should be tested for their microsatellite status. In MSI-H/dMMR CRC, checkpoint inhibitors should be used after therapy with fluoropyrimidine, oxaliplatin, and irinotecan. Currently, the value of checkpoint inhibitors in MSS CRC is still unclear.

### Gastroesophageal Cancer

Compared to CRC, the application of immunotherapy has been limited in gastroesophageal cancer due to tumor heterogeneity and complex immunosuppressive mechanisms. Checkpoint inhibition therapy showed some success in early trials, which is likely to be related to a specific phenotype of GC and its histopathology (162).

According to the outcomes of the KEYNOTE-059 trial, pembrolizumab has been approved by the FDA for the management of refractory GC. In addition, a series of KEYNOTE trials have been conducted in EC, including KEYNOTE-180, KEYNOTE-181, and KEYNOTE-590 (90, 163, 164). Nivolumab, based on the outcomes of ATTRACTION-2, was also approved in heavily pretreated tumors in Japan (165). Checkmate-032 confirmed that the combination of ipilimumab with nivolumab was superior to nivolumab monotherapy (166, 167). ATTRACTION-3 compared nivolumab to chemotherapy in refractory OC and demonstrated significant improvement in survival (median OS 10.9 months *vs*. 8.4 months; hazard ratio for death 0.77, p = 0.019) (168). Understanding the role of immunotherapy in GC has been improved with a shift in the classification of tumors, and four genomic subtypes of GC have been identified. Similar to CRC, the MSI-H population in GC has shown an encouraging response, as indicated by a subset analysis of trials (169). The EBV subtype is even more promising (170), with all patients of this subtype responding to pembrolizumab immunotherapy in a phase II trial (171). Meanwhile, there are still ongoing trials to evaluate the efficacy of different available immunotherapy agents, such as KEYNOTE-062 and KEYNOTE-061 (**Table 1**).

**Table 1:** Landmark trials of immunotherapy in GI.

Name of trial	Title	Interventions	Phases
**Colorectal Cancer**			
KEYNOTE 028	Nivolumab in patients with metastatic DNA mismatch repair-deficient or microsatellite instability-high colorectal cancer	Drug: Pembrolizumab	Phase 2
CheckMate 142	Nivolumab + ipilimumab combination in patients with DNA mismatch repair-deficient/microsatellite instability-high metastatic colorectal cancer	Drug: Nivolumab	Phase 2
CheckMate 142 (further analysis of subgroup)	Mismatch repair deficiency predicts response of solid tumors to PD-1 blockade	Drug: Nivolumab+ Ipilumumab	Phase 2
**Gastroesophageal Cancer**			
ATTRACTION-2 CheckMate-032	A study of nivolumab by itself or nivolumab combined with ipilimumab in patients with advanced or metastatic solid tumors	Drug: Nivolumab	Phase 1-2
KEYNOTE-012 KEYNOTE-059	A study of pembrolizumab in participants with recurrent or metastatic gastric or gastroesophageal junction adenocarcinoma	Drug: Pembrolizumab	Phase 2
NCT01585987	An efficacy study in gastric and gastroesophageal junction cancer comparing ipilimumab versus standard of care immediately following first line chemotherapy	Drug: Ipilimumab	Phase 2
NCT01693562	A phase I dose-escalation and cohort expansion study of lirilumab (anti-KIR; BMS-986015) administered in combination with nivolumab (anti-PD-1; BMS-936558; ONO-4538) in patients (Pts) with advanced refractory solid tumors.	Drug: MEDI4736	Phase 1-2

## Concluding Remarks

In summary, this review systematically describes immunological aspects of GI cancer and discusses the latest progress in cancer immunotherapy. Although immune-targeted therapy has gradually become the mainstream strategy for cancer treatment, side effects, drug resistance and a low response rate still represent challenges for researchers and clinicians. In addition, because immunotherapy may not benefit all the cancer types, there are some patient populations who cannot receive immunotherapy. Based on the above, we should strive to improve the understanding of tumor immunology, and the curative effect should be emphasized in further research. Over the next few decades, we expect to see the advent of more effective immunotherapy, the development of predictive biomarkers or more personalized treatment to help patients who are less likely to respond to current treatments to improve the treatment effect.

## Author Contributions

D-KW and QZ drafted the manuscript. BL and Q-YH were involved in design of the study and revision of the manuscript. All authors contributed to the article and approved the submitted version.

## Funding

This work was supported by National Natural Science Foundation of China (81973339, 31961160727, 81773085, 31770888) and the National Key Research and Development Program of China (2017YFA0505100).

## Conflict of Interest

The authors declare that the research was conducted in the absence of any commercial or financial relationships that could be construed as a potential conflict of interest.

## Publisher’s Note

All claims expressed in this article are solely those of the authors and do not necessarily represent those of their affiliated organizations, or those of the publisher, the editors and the reviewers. Any product that may be evaluated in this article, or claim that may be made by its manufacturer, is not guaranteed or endorsed by the publisher.

## Glossary

**Table:**

GEJ	gastric/esophagogastric junction
CRC	colorectal cancer
EC	esophageal cancer
GC	gastric cancer
ESCC	esophageal squamous cell carcinoma
SCC	esophageal adenocarcinoma
CRT	chemoradiotherapy
TNM	tumor node metastasis
CAR-T	chimeric antigen receptor-therapy
EGFR2	epidermal growth factor receptor 2
TAMs	tumor-associated macrophages
IFN	interferon
IL	interleukin
TCR	T cell receptor
BCR	B cell receptor
MHC	major histocompatibility complex
NK cell	natural killer cell
DCs	dendritic cells
CTLA-4	cytotoxic T-lymphocyte-associated protein 4
LAG-3	lymphocyte activation gene 3
ASCO	American Society of Clinical Oncology
TIM-3	T cell immunoglobulin 3
PD-1	programmed cell death 1
PD-L1	programmed cell death ligand 1
ACT	adoptive cell therapy
NKT cell	natural killer T cell
IDO	indoleamine 2,3-dioxygenase
MSI	microsatellite instability
MSS	microsatellite stability
dMMR	defective match repair
pMMR	proficient match repair
ORR	objective response rate
EBV	Epstein-Barr virus
APC	antigen-presenting cell
CMS 1-4	consensus molecular subtype 1-4
TGF-β	transforming growth factor β
TNF-α	tumor necrosis factor α
GM-CSF	granulocyte-macrophage colony stimulating factor
Mst1	macrophage stimulator 1
CCL2/CCR2	C-C motif chemokine ligand 2/C-C motif chemokine ligand 2 receptor
CSF1R	colony stimulating factor-1 receptor
KIRs	killer-cell immunoglobulin-like receptors
NKG2A	CD94/NK group 2 member A
HLA	leukocyte antigen
TME	tumor microenvironment
TIME	tumor immune microenvironment
MDSCs	myeloid derived suppressor cells
CDR3	complementarity determining region 3
ADCP	antibody-dependent cellular phagocytosis
CEA	carcino-embryonic antigen
HER2	human epidermal growth factor receptor-2
GPC3	complement C3 protein
EpCAM	epithelial cell adhesion molecule
EGFR	epidermal growth factor receptor
VEGF	vascular endothelial growth factor
SIRPα	signal regulatory protein-α
PLR	platelet-lymphocyte ratio
TAP2	ATP binding cassette subfamily B member 3
ICI	immune checkpoint inhibitor therapy
ECM	extracellular matrix
TILs	tumor infiltrating lymphocytes
CAFs	cancer-associated fibroblasts
EGF	epidermal growth factor
Breg cells	regulatory B cells
Treg cells	regulatory T cells
RCC	renal cell carcinoma
CEA	carcinoembryonic antigen
ROS	reactive oxygen species
RNS	reactive nitrogen species
MMP9	matrix metalloproteinase 9
NETs	neutrophil extracellular traps
MSI-H	microsatellite instability-high
iNKT cells	invariant natural killer T cells
PFS	progression-free survival
OS	overall survival
